# Rationale, design, and protocol for the prevention of low back pain in the military (POLM) trial (NCT00373009)

**DOI:** 10.1186/1471-2474-8-92

**Published:** 2007-09-14

**Authors:** Steven Z George, John D Childs, Deydre S Teyhen, Samuel S Wu, Alison C Wright, Jessica L Dugan, Michael E Robinson

**Affiliations:** 1 Department of Physical Therapy, Brooks Center for Rehabilitation Studies, PO Box 100154, University of Florida, Gainesville, FL, 32610-0154, USA; 2 US Army-Baylor University Doctoral Program in Physical Therapy (MCCS-HMT), Army Medical Department Center and School, 3151 Scott Rd., Rm. 2307, Fort Sam Houston, TX 78234, USA; 3 Department of Epidemiology and Health Policy Research, PO Box 100177, University of Florida, Gainesville, FL 32610-0177, USA; 4 Department of Clinical and Health Psychology, Center for Pain Research and Behavioral Health, University of Florida, Gainesville, FL 32610-0165, USA

## Abstract

**Background:**

There are few effective strategies reported for the primary prevention of low back pain (LBP). Core stabilization exercises targeting the deep abdominal and trunk musculature and psychosocial education programs addressing patient beliefs and coping styles represent the current best evidence for secondary prevention of low back pain. However, these programs have not been widely tested to determine if they are effective at preventing the primary onset and/or severity of LBP. The purpose of this cluster randomized clinical trial is to determine if a combined core stabilization exercise and education program is effective in preventing the onset and/or severity of LBP. The effect of the combined program will be compared to three other standard programs.

**Methods/Design:**

Consecutive Soldiers participating in advanced individual training (AIT) will be screened for eligibility requirements and consented to study participation, as appropriate. Companies of Soldiers will be randomly assigned to receive the following standard prevention programs; a core stabilization exercise program (CSEP) alone, a CSEP with a psychosocial education (PSEP), a traditional exercise (TEP), or a TEP with a PSEP. Proximal outcome measures will be assessed at the conclusion of AIT (a 12 week training period) and include imaging of deep lumbar musculature using real-time ultrasound imaging and beliefs about LBP by self-report questionnaire. We are hypothesizing that Soldiers receiving the CSEP will have improved thickness of selected deep lumbar musculature (transversus abdominus, multifidi, and erector spinae muscles). We are also hypothesizing that Soldiers receiving the PSEP will have improved beliefs about the management of LBP. After AIT, Soldiers will be followed monthly to measure the distal outcomes of LBP occurrence and severity. This information will be collected during the subsequent 2 years following completion of AIT using a web-based data entry system. Soldiers will receive a monthly email that queries whether any LBP was experienced in the previous calendar month. Soldiers reporting LBP will enter episode-specific data related to pain intensity, pain-related disability, fear-avoidance beliefs, and pain catastrophizing. We are hypothesizing that Soldiers receiving the CSEP and PSEP will report the longest duration to first episode of LBP, the lowest frequency of LBP, and the lowest severity of LBP episodes. Statistical comparisons will be made between each of the randomly assigned prevention programs to test our hypotheses related to determining which of the 4 programs is most effective.

**Discussion:**

We have presented the design and protocol for the POLM trial. Completion of this trial will provide important information on how to effectively train Soldiers for the prevention of LBP.

**Trial registration:**

NCT00373009

## Background

Low back pain (LBP) is one of the most common forms of chronic pain [1,2] and is a significant cause of disability and cost in society [3-6]. Chronic LBP substantially influences the capacity to work and has been associated with the inability to obtain or maintain employment [5] and lost productivity [6]. Specific to the United States military, LBP was the second most common reason to seek healthcare and affects over 150,000 active duty Soldiers annually [7]. Soldiers in the U.S. Army with LBP have the highest risk of disability 5 years after their injury [8], and LBP was the most common condition bringing about a medical board, with lifetime direct compensation costs estimated to reach into the billions of dollars [9]. Reduction of disability from LBP is a significant research priority for the military.

Reduction of disability from LBP has been divided into 2 separate phases – primary and secondary prevention. Primary prevention refers to interventions and strategies that are implemented before a low back injury occurs [10]. Primary prevention reduces LBP related disability by reducing the total number of people who eventually experience an episode of LBP. Secondary prevention refers to interventions and strategies that are implemented during the acute episode of low back injury, before chronic symptoms occur [11]. Secondary prevention reduces LBP related disability by reducing the number of people who eventually experience chronic disability from LBP. This cluster randomized clinical trial incorporates a combination of primary and secondary prevention strategies for limiting the occurrence and severity of LBP for active duty Soldiers in the U. S. Army.

### Primary prevention

Theoretically, primary prevention would be the most effective manner to reduce disability from LBP; however, the current scientific literature does not support commonly used methods. For example, randomized clinical trials in occupational settings have demonstrated the ineffectiveness of commonly used primary prevention strategies such as back schools, lumbar supports, and ergonomic interventions [12,13]. Despite this lack of evidence, efforts continue to investigate primary prevention interventions because of the obvious benefits of reducing LBP before it occurs. A recent review article suggests that future research related to primary prevention should focus on exercise programs, as they may offer the greatest potential for reducing disability from LBP [12]. Core stabilization exercise programs (CSEP) may be a good choice for primary prevention studies because biomechanical, anatomical, and clinical studies provide evidence that core stabilization is an effective intervention [14-16].

### Biomechanical and anatomical evidence supporting core stabilization

Core muscles attached to the spine such as the transversus abdominus, multifidus, and the erector spinae play a key supportive role that contribute to the ability of the lumbar spine to withstand loading [17,18]. As an example, the transversus abdominus, one of the deep abdominal muscles, stabilizes the spine by forming a corset or rigid cylinder around the spine. Recent evidence supports a feed-forward postural control role for the transversus abdominus as it relates to limb movement [19-23]. Hodges et al [19-21,23] demonstrated that transverses abdominus muscle activation occurred prior to limb movement (regardless of directions) in asymptomatic adults. However, in patients with LBP, there is a delay in activation of transversus abdominus contraction relative to the primary muscles of the limb [24-26], suggesting that people with LBP lack optimal stability of the spine prior to activities requiring limb movements.

The multifidi are small intrinsic muscles that function as the primary intersegmental stabilizers of the spine [17]. Poor endurance of the multifidus is a predictor of increased recurrence of LBP. Further, multifidus atrophy and decreased muscular activity tends to occur on the side of symptoms [27-29]. The magnitude of atrophy has also been linked with poor outcomes following laminectomy surgery [30]. Furthermore, the multifidi do not automatically recover full strength and endurance after the first episode of LBP unless specific rehabilitation is done [31]. Hides et al [15,27,31] demonstrated that patients with >30% discrepancy in the cross-sectional area of the multifidus muscle are at an increased risk for having recurrent LBP unless treated with a CSEP.

The erector spinae (longissimus thoracis and iliocostalis lumborum) primarily produce extensor force needed for lifting but also stabilizes the spine. McGill has shown that the pars lumborum portions of these muscles are able to produce significant torque moments around all three orthopedic axes of motion [32], while Cholewicki demonstrated an antagonistic co-activation of trunk flexors and extensors occurs around the neutral spine in healthy subjects [33]. This co-activation increased in response to addition of an external load. In addition, Lee et al [34] found that, in a cohort of subjects followed for five years, the development of LBP was associated with lower levels of extensor strength compared to flexor strength. The convergence of these findings supports the need to further examine the effectiveness of exercise programs that target these muscles in preventing LBP.

### Clinical evidence supporting core stabilization

Treatment and prevention exercise programs for LBP that have been reported in the literature commonly involve muscles involved in core stabilization such as the transversus abdominus, multifidi, and erector spinae muscles [15,16,35]. The fundamental component of these exercise programs is that they improve the neuromuscular activation and control of the targeted muscles. Reports in the literature have also highlighted that these programs may be an effective way to reduce disability from LBP. For example, a randomized clinical trial demonstrated that performance of a CSEP emphasizing activation of the transversus abdominus caused fewer recurrences of LBP 3 years following treatment for first time LBP [15]. Individuals with a >30% discrepancy in the cross-sectional area of the multifidus who completed a specific CSEP experienced 50% fewer recurrences of LBP at one year and 40% fewer recurrences at three years after treatment compared to individuals who received standard of care medical treatment [15].

The lack of core stabilization has been identified as a potential predictor of an individual's risk of developing recurrent LBP [36,37], further increasing the impetus for incorporating CSEP into routine physical training programs across the United States Army. While these assertions regarding CSEP and LBP prevention are promising, they have not been rigorously tested in clinical trials involving healthy Soldiers. Differences in muscle training are important to consider because TEP training focuses on muscles (i.e. rectus abdominus and oblique abdominals) not consistently supported by biomechanical and anatomical evidence [19,24,38,39]. In fact, a clinical trial suggests that exercises included in a TEP were ineffective at preventing LBP [40]. Therefore, it is not known if performance of CSEP effectively prevents LBP when compared to a traditional exercise program (TEP) commonly implemented in physical training.

### Secondary prevention

The scientific literature has also investigated secondary prevention as a strategy to reduce disability from LBP because effective primary prevention strategies are currently lacking [11]. Secondary prevention strategies have met with some success, and two consistent themes have developed. The first theme is that psychological factors play a significant role in the development of chronic disability from LBP. Prospective studies involving patients with acute LBP have consistently demonstrated that when compared to demographic or physical factors, psychological factors are the strongest predictors of chronic disability from LBP [41,42]. The second theme is that early interventions that address these psychological factors result in decreased disability from LBP [43-45].

### Psychological model for the development of chronic low back pain

Psychological models are commonly used to explain one manner in which chronic disability develops from LBP [46,47] and one specific model is the Fear-Avoidance Model (FAM) [48]. This model proposed that *fear-avoidance beliefs *and *pain catastrophizing *are the primary psychosocial factors involved in the development and maintenance of chronic symptoms. Fear-avoidance beliefs are comprised of an individual's pain experiences, present stress level, pain behavior, and certain personality traits [49]. Fear-avoidance beliefs detail an individual's fear of pain and re-injury specific to LBP and the belief as to whether physical activity should be maintained while experiencing LBP [49]. Pain catastrophizing is a negative, multidimensional construct comprised of rumination, helplessness, and pessimism cognitions [50]. Pain catastrophizing is related to the belief that the experienced pain will inevitably result in the worst possible outcome [50].

Collectively, these psychosocial factors determined the response to an episode of LBP along a continuum from confrontation to avoidance. A confrontation strategy (low levels of fear-avoidance beliefs and pain catastrophizing) is viewed as an adaptive response, enabling the individual to return to normal vocational and social activities. An avoidance strategy (high levels of fear-avoidance beliefs and pain catastrophizing) is viewed as a maladaptive response. The consequences of an avoidance strategy are theorized to be both psychological (hyperalgesia) and physical (chronic disability and reductions in physical performance). Furthermore, continuation of an avoidance response contributes to the pain experience in a deleterious manner by making it more likely to maintain high levels of pain-related fear and pain catastrophizing.

### Clinical evidence supporting psychosocial education programs

Fear-avoidance beliefs and pain catastrophizing were strongly associated with pain intensity and disability in patients with chronic LBP [51-55]. Longitudinal studies have demonstrated that fear-avoidance beliefs and pain catastrophizing are also precursors to the development of chronic disability [41,56-58]. As a result, it has been hypothesized that early reduction of fear-avoidance beliefs and pain catastrophizing is an important way to reduce development of chronic LBP.

Psychosocial education programs (PSEP) that reduce fear-avoidance beliefs and pain catastrophizing have been described in the literature [44,45,59,60]. These educational programs differ from traditional educational approaches in that they de-emphasize the anatomical cause of LBP (as it often cannot be determined), encourage the patient to take an active role in his recovery, provide evidence-based information on LBP management and outcome, teach the patient to view back pain as a common (i.e. not a serious disease) condition, and instruct the individual on the importance of maintaining positive attitude and coping styles (i.e. limiting fear-avoidance beliefs and pain catastrophizing).

Randomized clinical trials and quasi-experimental designs provide consistent evidence that PSEP's decrease maladaptive beliefs and coping styles in healthy individuals and patients experiencing LBP [43,44,61,62]. Furthermore, early evidence from randomized clinical trials suggests that psychological and physical LBP severity (i.e. fear-avoidance beliefs, coping strategies, pain intensity and/or pain-related disability) can be decreased when PSEP's are implemented in individuals experiencing LBP [43-45]. This evidence is promising, as it suggests that severity of LBP can be favorably modified with a PSEP. Although a PSEP delivered via public service announcements has been investigated in healthy individuals and found to decrease beliefs associated with LBP [62], no research has determined if PSEPs are effective at reducing the occurrence or severity of LBP when administered to healthy individuals.

### Summary and purpose

The accumulated evidence supports the potential of CSEP and PSEP for prevention of LBP. Early evidence supports the effectiveness of these combined programs for reducing future disability in patients already experiencing LBP [44,45]. However, the effect of early implementation (i.e. in healthy individuals before the onset of LBP) of combining CSEP and PSEP has not been previously investigated in a large-scale, controlled study. The purpose of the Prevention of Low Back Pain in the Military (POLM) trial is to determine if a combination of CSEP and PSEP is effective in limiting the onset of LBP and/or the severity of LBP. The effect of this combined program will be compared to three other standard programs.

## Methods/Design

**Figure 1:**
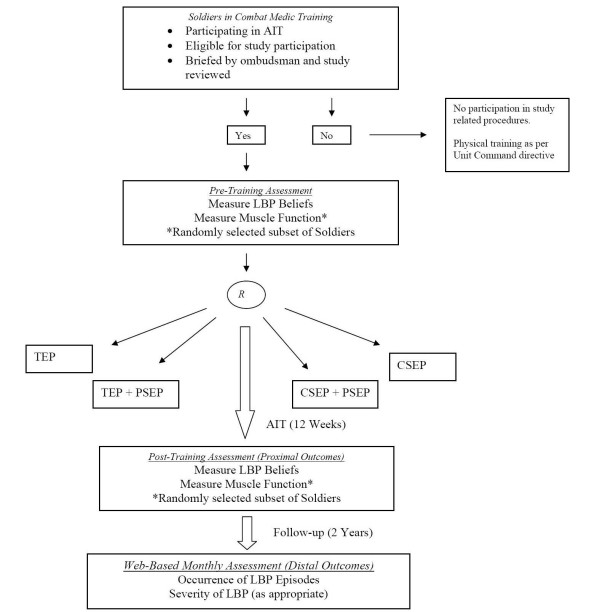
**Proposed study design of the prevention of low back pain in the military (POLM) trial**. LBP, low back pain; AIT, advanced individual training; CSEP, core stabilization exercise program; PSEP, psychosocial education program; TEP, traditional exercise program.

The institutional review boards at the Brooke Army Medical Center (Fort Sam Houston, Texas) and the University of Florida (Gainesville, FL) have granted approval for this project. Figure 1 provides an overview of the proposed study design.

### Subjects

We will be recruiting consecutive Soldiers entering the combat medic 12-week advanced individual training (AIT) program at Fort Sam Houston, Texas. Soldiers will be screened for eligibility according to the following inclusion/exclusion criteria:

#### Inclusion criteria

• Ages 18 (or emancipated minor that is 17 years old) – 35 years old

• Participating in combat medic military occupational specialty (MOS) training

• English speaking and reading

#### Exclusion criteria

• Prior history of LBP (defined as LBP that limited work or physical activity for greater than 48 hours or caused individual to seek healthcare)

• Currently seeking medical care for LBP

• Previous medical history including history of degenerative joint disease, arthritis, spine trauma or vertebral fractures, spondylolisthesis, and/or congenital spine disorders. This also includes any prior surgery for LBP

• Currently unable to participate in unit physical training due to injury in foot, ankle, knee, hip, neck, shoulder, elbow, wrist, or hand injury.

• History of fracture (stress or traumatic) in proximal femur and/or pelvis

• Currently pregnant (later pregnancy will not result in termination from the study, but it is an exclusion criteria at enrollment.)

• Have been rolled over from another Company participating in combat medic military occupational specialty (MOS) training

Research staff at Fort Sam Houston, Texas will introduce this study to Soldiers, screen them for eligibility, and obtain informed consent from Soldiers, as appropriate. The informed consent document will obtain permission from Soldiers to perform the study-related procedures and to contact them at their civilian address if any of the participants have been separated or discharged from active duty during the 2-year follow-up period. After providing informed consent, Soldiers will be issued a card with username, password, and information for accessing a secure website hosted by the University of Florida. Subjects will be monitored through all four stages of this trial (enrollment, intervention allocation, follow-up, and data analysis) in compliance with CONSORT guidelines [63]. For example, we will record reasons for a subject dropping out of the study during any stage of the trial and we will record all reasons for non-participation in the study to enable our ability to calculate an overall participation rate.

### Randomization

We acknowledge the ideal study design to answer our research question would involve individual randomization of Soldiers to the 4 prevention programs. However, individual randomization presents unique challenges in a military training environment that would seriously impede this study's feasibility. Military training environments require Soldiers to live in close quarters with other members of their unit, to facilitate optimal training and to foster esprit de corps. Soldiers function in teams during many of their training activities, including unit physical training. Specifically, we elected not to individually randomize to the prevention programs because a) it would potentially detract from unit cohesion, b) contamination of the treatment groups would be inevitable, and c) the administration of the study would be excessively burdensome for drill instructors leading unit physical training.

Therefore, we will utilize a cluster randomization strategy by randomly assigning company units, such that every Soldier in the company who consents to participation in the study completes the same prevention program. Cluster randomization has been effectively used in other investigations involving large samples of musculoskeletal injury prevention, adherence to quality indicators for prevention of cardiovascular disease, and the effect of a community-based intervention on maternal depression [64-66]. The cluster randomization schedule will be determined before recruitment begins and will be balanced.

### Exercise programs

The Soldiers' drill instructors will receive comprehensive training in the study procedures prior to beginning the study to insure their proficiency in administering the standardized exercise programs. Drill instructors will be issued detailed pre-prepared training cards specific to each program and training information will also be available on a study-related web-site. These training cards will be used to ensure the proper administration of the training protocol for a particular company. Study personnel will be present at training times to ensure compliance with the assigned exercise program.

#### Traditional exercise program (TEP)

The TEP was selected from commonly performed exercises for the rectus abdominus and oblique abdominal muscles. These exercises are traditionally performed in the military environment and are commonly utilized to assess physical performance of Soldiers. Soldiers will be instructed to perform the exercises in a group setting under the direct supervision of their drill instructor. This exercise regiment consists of 5 exercises and each will be performed for 1 minute. The TEP will be performed daily, for a total dosage time of 5 minutes/day, 5 days per week. Having Soldiers perform the TEP in group physical training settings will help ensure compliance with the TEP.

Exercises in the TEP are widely utilized inside (and outside) the military for physical training purposes. These exercises target the rectus abdominus and oblique abdominal muscles, which are not supported by the accumulated anatomical, biomechanical, and clinical evidence for preventing LBP [15,24,27,31,38,40]. Furthermore, the exercise prescription emphasizes quick activation, high load, and high repetitions with full movements of the trunk and this type of prescription does not match these muscles' function [38,67]. We believe the TEP will not effectively prevent LBP because it focuses on trunk musculature not highlighted in the LBP prevention literature and exercises muscles in a sub-optimal manner.

#### Core stabilization exercise program (CSEP)

The CSEP will consist of exercises from the accumulated evidence shown to selectively activate the transversus abdominus, multifidi, and erector spinae. Soldiers in this group will perform crunches in lieu of regular sit-ups. Soldiers will be issued photographs of the exercises with written instruction in technique. Then, Soldiers will be instructed to perform the exercises in a group setting under the direct supervision of the drill instructor. This exercise regiment consists of 5 exercises and each will be performed for 1 minute. The CSEP will be performed daily, for a total dosage time of 5 minutes per day, 5 days per week. Having Soldiers perform the CSEP in-group physical training settings will ensure compliance with the CSEP.

The CSEP was selected from current evidence previously discussed. [15,24,27,31,38,40] This literature demonstrates that these exercises increase activation of key core musculature. The exercise prescription for the CSEP follows a slow activation, low load principle with minimal trunk movements, that best matches these muscles' function, according to noted experts in the area [38,67] These exercises are also supported by the United States Army Physical Fitness Program's new doctrine, yet they have not been clinically tested for preventing LBP. We hypothesize the CSEP will effectively prevent LBP because it focuses on core musculature highlighted in the LBP prevention literature and exercises these muscles in an appropriate manner. Table 1 provides a brief comparison of the two exercise programs.

**Table 1:** Comparison of traditional (TEP) and core stabilization exercise (CSEP) programs

**Exercise**	**CSEP**	**TEP**
**Principle**	Lower load, less repetitions	Higher load, more repetitions
**Activation**	Slower	Faster
**Trunk movements**	None to minimal	Full
**Dosage**	5 minutes/day	5 minutes/day
#1	Abdominal drawing-in maneuver crunch	Traditional sit-up
#2	Left and right horizontal side support	Sit-up with left trunk rotation
#3	Hip flexor squat ('wood-chopper')	Sit-up with right trunk rotation
#4	Supine shoulder bridge	Abdominal crunch
#5	Quadruped alternate arm and leg	Traditional sit-up

TEP, traditional exercise program; CSEP, core stabilization exercise program

### Psychosocial education program (PSEP)

We elected not to include a traditional education program in this trial, as prior studies consistently demonstrate traditional education does not favorably change LBP beliefs[44,61,62,68] The education program involves attending 1 educational session during the first week of AIT for randomly assigned soldiers. The session will involve an interactive seminar led by study personnel lasting approximately 45 minutes. The seminar will consist of a visual presentation that presently comprises evidence-based education for LBP.

The seminar will cover topics like the prognosis of LBP, stressing that anatomical causes of LBP are not likely, and emphasizing the importance of decreasing fear-avoidance beliefs and pain catastrophizing in response to LBP. Educational material about the natural course of low back pain will be included. After the seminar, Soldiers will be involved in a question and answer session led by study personnel. Finally, Soldiers will be issued *The Back Book *for their personal use. *The Back Book *is being used because we have prior experience with it and it has been demonstrated to reduce fear-avoidance beliefs [43,44]. Proper administration of the PSEP will be ensured by having study personnel lead the educational session for Soldiers assigned to receive PSEP.

### Blinding

It is not possible to mask Soldiers in this study because they will actively participate in the randomly assigned training programs. Post-training physical examinations and real time ultra-sound imaging will be performed by personnel unaware of program assignment. Soldiers will be instructed not to discuss their group assignment with study personnel during post-training examinations unless there is an urgent reason to do so (e.g. for medical reasons).

### Measures

Study related measures are separated into proximal outcome measures, consisting of self-report and physical measures (pre and post AIT), and distal outcome measures, consisting of LBP episode-related measures (2 years active duty).

#### Proximal outcome – self-report measures (pre and post training)

##### Physical and Mental Function

The Medical Outcomes Survey 12-Item Short-Form Health Survey (SF-12) will be used as a self-report of health status for physical and mental function. The derived physical component summary scale (PCS) and mental component summary (MCS) are believed to be a valid option to represent the eight domains of physical and mental components of health [69].

##### Negative Affect

The State-Trait Anxiety Questionnaire (STAI) will be used to measure negative affect from anxiety [70]. The Beck Depression Inventory (BDI) will be used to measure negative affect from depression [71-73].

##### Fear of Pain

The Fear of Pain Questionnaire (FPQ-9) will be used to measure fear about specific situations that normally produce pain [74-76].

##### LBP Beliefs

The Back Beliefs Questionnaire (BBQ) will be used to beliefs about management and outcome associated with LBP [61,77].

#### Proximal outcome – physical measures (pre and post training)

Randomly selected Soldiers (n = 20) from each company will undergo physical measures. This decision was made due to the time and expense associated with performing a physical examination and real-time ultrasound imaging on a sample this large.

##### Physical Impairment

Total lumbar flexion and straight leg raise from the physical impairment scale described by Waddell et al [78] will be used in this study. Range of motion measurements of bilateral hip internal and external rotation will be used. Additionally, 4 trunk endurance tests will be used for maintaining extensor, flexor, and bilateral side support positions.

##### Real-Time Ultrasound Imaging

All real-time ultrasound measurements of the deep trunk muscles will be obtained using a Sonosite 180 Plus (Sonosite Inc. Bothell, WA) with a 5 MHz curvilinear array for the lateral abdominal muscles and the posterior trunk muscles [79]. Ultrasound measurements of the lateral abdominal muscles (transversus abdominus, internal oblique, and external oblique) during the active straight leg test maneuver will be obtained following the techniques outlined by Teyhen et al [80] Symmetry measurements of the multifidi muscles will be performed as outlined by Hides et al [81].

#### Distal outcome – low back pain (LBP) episode-related measures

We will follow Soldiers for 2 years following graduation from AIT to record the number and the severity of LBP episodes experienced. Monthly emails containing a link to the University of Florida hosted POLM website will query Soldiers on whether they have experienced any LBP in the last calendar month, and if so, the Soldiers will be prompted to complete the information described below.

##### Compliance

Compliance to the Soldiers' randomly assigned exercise and education programs will be recorded for each month.

##### Onset of LBP

Soldiers will be queried whether they have experienced LBP in the last calendar month. If they have, Soldiers will be cued to answer following validated self-report questionnaires.

##### Disability

Self-report of low back-related disability will be assessed with the Oswestry Disability Questionnaire (ODQ), a scale originally described by Fairbank et al [82]. The ODQ that will be used in this study was modified from the original version by substituting a section regarding employment/home-making ability for the section related to sex life [83,84].

##### Pain

Patients will rate their pain intensity and unpleasantness using a numerical rating scale (NRS). The NRS consist of 11 points whose endpoints are designated as '0 – no pain sensation' and '10 – the most intense pain sensation imaginable.'

##### Fear-Avoidance Beliefs

The Fear-Avoidance Beliefs Questionnaire (FABQ) will be used to quantify fear-avoidance beliefs in this study [53].

##### Pain Catastrophizing

The Pain Catastrophizing Scale (PCS) will be used to quantify pain catastrophizing [85].

### Data analysis

Demographic and baseline levels of clinical variables will be compared between the 4 cluster randomized groups using analysis of variance (ANOVA) for comparison of means and chi-square tests for comparison of proportions. Variables found to be significantly different between the training groups will be considered in the final analyses, in addition to prespecified covariates (gender, age, and physical impairment). Six analyses will be performed based on our pre-specified hypotheses. Primary outcomes will be analyzed with Poisson regression for occurrence of LBP and Cox regression for time to first episode of LBP. Secondary outcomes will be analyzed with MANOVA and ANOVA models. Based on Bonferroni adjustment, we will conduct each of the hypothesis tests two-sided at the 0.008 levels. All statistical analyses will be performed using the SAS software, version 9 (SAS Institute Inc, 1996).

#### Sample size estimation and power analysis

Company size and consent rate are expected to vary, so the following represent the assumptions used for a sample size estimation and power analysis. A total of 16 companies could potentially be randomly assigned to the 4 programs, with approximately 200 eligible Soldiers per company, and 75% are expected to provide consent for study participation. Our sample size estimation was based on determining the effect of the CSEP and PSEP on preventing the occurrence and severity of LBP episodes.

We expect that 33% Soldiers performing a prevention program will experience LBP compared to 51% for those in the control group [86]. For a group difference of such magnitude, a two-sided statistical test at 0.008 level should have more than 99% of power for 4 companies of soldiers. However, we will enroll up to 16 companies because only the Soldiers reporting LBP will be included in certain hypotheses. With 16 companies, we expect approximately 450 soldiers in the combined program and 675 soldiers in traditional program group to experience LBP. Data in Table 2 demonstrates the expected power to detect differences among Soldiers experiencing LBP using pilot estimates from George et al [44].

**Table 2:** Power estimates for low back pain episode specific outcomes

**Measure of LBP severity**	**Traditional program**	**Combined program**	**Power (16 companies)**	**Power (12 companies)**
FABQ (physical activity)	13.5 (sd = 7.0)	10.1 (sd = 5.9)	100%	100%
FABQ (work)	12.3 (sd = 12.3)	9.7 (sd = 10.2)	86%	65%
ODQ	15.5 (sd = 17.9)	11.9 (sd = 10.0)	92%	77%

FABQ, Fear-avoidance beliefs questionnaire; ODQ, Oswestry disability questionnaire

For our proximal outcomes, we will randomly sample Soldiers from each company. These Soldiers will be assessed by physical examination and with real-time ultrasound imaging to measure changes in specific core muscles during AIT. Our assumptions for power calculations were that statistical tests will be conducted at the 0.007 levels and we conservatively assumed that the differences in specific core musculature between Soldiers completing CSEP and those not completing CSEP in this study would be at least half the amount seen in the pilot estimates from Teyhen et al [80]. A sample size of 16 companies will provide more than 90% power as shown in Table 3.

**Table 3:** Power estimates for muscle imaging outcomes

**Muscle measure (thickness)**	**TEP**	**CSEP**	**Power (16 Companies) **
Transversus abdominus	1.5 (sd = 0.5)	2.0 (sd = 0.5)	100%
Erector spinae	31.0 (sd = 6.0)	34.5 (sd = 6.0)	90%
Multifidi (symmetry)	20.0% (sd = 6.0)	11.5% (sd = 6.0)	100%

TEP, traditional exercise program; CSEP, core stabilization exercise program

#### Treatment of Soldiers not completing training protocol

There is approximately a 20% attrition rate for Soldiers not completing AIT. The reasons for attrition are varied, but can be broadly defined into medical, physical, personal, academic, or behavioral categories. Decisions regarding Soldier attrition are made by Commanding Officers, independent of the study investigators. Therefore, we have no direct influence on Soldier attrition rates. The consequence of attrition for the Soldier is that he or she joins another company and resumes AIT. The consequence of attrition for the proposed analysis plan is that the reassigned Soldier will likely be performing a different training protocol than original assigned. Therefore, such soldiers represent a potential internal validity threat to this study.

The following a priori decisions have been made to account for Soldiers that consented to study participation, but did not complete AIT. First, any Soldier completing less than 10-weeks of AIT will have the reason for attrition recorded, and will not be followed during active duty. Second, the reasons and rates of attrition will be compared between the 4 cluster randomized groups using chi-square tests for comparison of proportions. This approach will allow the investigators to protect the internal validity of the study, by ensuring Soldiers receiving multiple interventions of unknown duration are not followed during active deployment. This approach will also allow the investigators to determine if the attrition rates were consistent across companies throughout the length of the study.

#### Treatment of missing data

We will handle missing data values with a 3-step process. First, the dropout rates will be compared across the programs to assess systematic differences. Second, demographic and dependent variables will be examined for their relationship to dropout. Those variables related to dropout status will be used to impute missing values for use in the analyses described below (via Missing Items Analysis). This multiple imputation approach will be compared to a last observation carried forward approach, mixed models approach, or worst-case approach to missing data. In addition, we will analyze completers only, as a liberal estimate of treatment efficacy. Finally, comparison of the completers vs. imputation analyses will yield an additional estimate of the effect of dropouts on hypothesis tests.

## Discussion

We have presented the design and protocol for the POLM trial. We will train Soldiers with specific exercise and education programs and measure the occurrence and severity of LBP episodes over a 2-year period. Completion of this trial will provide important information on how to effectively train U.S. Soldiers for the prevention of LBP. Results of the POLM trial will be disseminated as soon as they are available.

## Abbreviations

LBP, low back pain; AIT, advanced individual training; CSEP, core stabilization exercise program; PSEP, psychosocial education program; MOS, military occupational specialty; POLM, Prevention of low back pain in the military trial; FAM, Fear-avoidance model; TEP, Traditional exercise program; STAI, State-trait anxiety questionnaire; FPQ, Fear of pain questionnaire; BBQ, Back beliefs questionnaire; SF-12, Medical outcomes survey 12-item short-form health survey; PCS, Physical component summary scale; MCS, Mental component summary scale; ODQ, Oswestry disability questionnaire; NRS, numerical rating scale; FABQ, Fear-avoidance beliefs questionnaire; PCS, Pain catastrophizing scale

## Competing interests

The author(s) declare that they have no competing interests.

## Authors' contributions

All authors read, edited, and approved the final version of the manuscript. SZG, JDC, DST, SSU, and MER were responsible for the initial conception of the research question, securing funding, supervising the protocol, and manuscript preparation. ACW and JLD were responsible for implementing study protocol and critically reviewing earlier versions of this manuscript.

## Pre-publication history

The pre-publication history for this paper can be accessed here:


